# Effects of hip abducted position during eccentric-only resistance exercise on acute changes in passive stiffness of the biarticular hamstring muscles

**DOI:** 10.1371/journal.pone.0338826

**Published:** 2025-12-18

**Authors:** Raki Kawama, Katsuki Takahashi, Ryo Nobukawa, Taku Wakahara

**Affiliations:** 1 Organization for Research Initiatives and Development, Doshisha University, Kyoto, Japan; 2 Faculty of Health and Sports Science, Doshisha University, Kyoto, Japan; 3 Human Performance Laboratory, Waseda University, Saitama, Japan; Nishikyushu University: Nishikyushu Daigaku, JAPAN

## Abstract

Recent studies have revealed that eccentric-only resistance exercise has the potential to acutely decrease passive stiffness of some biarticular hamstring muscles. The biarticular hamstring muscles are hip adductors and thus are greatly lengthened in the hip abducted position. Based on these findings, this study aimed to investigate whether eccentric-only resistance exercise in hip abducted positions, rather than in the hip-neutral position, induces a greater acute decrease in the passive stiffness of the biarticular hamstring muscles. Eleven participants performed eccentric-only knee flexions with different hip abducted positions (0° abduction [ABD0]; 20° abduction [ABD20]; 40° abduction [ABD40]) on separate days. Maximal joint range of motion, passive torque, shear moduli of the biarticular hamstring muscles, and maximal isometric torque were measured before and 5 min after completing each exercise. The shear moduli of the biceps femoris long head and semitendinosus were significantly lower at 5 min post-exercise than at pre-exercise in all hip joint positions. In the semimembranosus, the shear modulus was significantly lower at 5 min post-exercise than at pre-exercise at ABD0 and ABD20, but not at ABD40. In the three muscles, there were no significant differences in the changes in the shear moduli at 5 min post-exercise among the hip abducted positions. These findings suggest that eccentric-only resistance exercise in hip abducted positions does not necessarily amplify the acute decrease in the passive stiffness of the biarticular hamstring muscles and may even attenuate the decrease in stiffness of a specific muscle.

## Introduction

Passive muscle stiffness is an important component that relates to physical performance [1,2] and injury susceptibility [3]. Studies have shown that individuals with higher passive muscle stiffness (shear modulus assessed by ultrasound shear wave elastography) have lower performance of long-distance running [1] and a higher risk of musculoskeletal injuries [3]. Given these functional and clinical relevance, chronically decreasing passive muscle stiffness has been a topic of considerable interest for some practitioners. Independent studies suggest that chronic decreases in passive muscle stiffness [4,5] may be achieved by repeatedly performing static stretching and eccentric contractions that immediately decrease muscle stiffness following a single session [6,7], although this association has not yet been directly examined within the same participants. Thus, an understanding of the acute change in muscle stiffness could provide insights into its chronic change.

One of the most common approaches to acutely decrease passive muscle stiffness is performing stretching exercises. Several studies have reported immediate decreases in passive muscle stiffness after one session of static stretching [6,8]. Meanwhile, recent studies revealed that passive muscle stiffness also acutely decreased after eccentric-only resistance exercises [7,9,10]. For example, the shear moduli of the biceps femoris long head (BFlh) and the semimembranosus (SM) decreased immediately after eccentric-only knee flexion [10] and hip extension [7,9], respectively. These findings provide novel approaches to acutely decrease passive muscle stiffness, but the magnitude of the decrease in the shear moduli of some of the biarticular hamstring muscles was substantially smaller in the studies using eccentric-only resistance exercise (approximately −5.0 to −0.8 kPa in BFlh, −4.5 to −0.6 kPa in the semitendinosus [ST], −10.7 to −7.2 kPa in SM [7,9]) than in the stretching study (−12.9 kPa in BFlh, −6.6 kPa in ST, −34.0 kPa in SM [6]). Thus, further approaches are necessary to achieve a greater acute decrease in passive muscle stiffness by eccentric-only resistance exercise.

One potential approach to greatly decrease passive stiffness of the biarticular hamstring muscles is to modify the hip joint position during resistance exercise. The biarticular hamstring muscles cross the hip and knee joints. The hip joint, being a ball-and-socket joint, allows three-dimensional motions, such as adduction/abduction and internal/external rotation in addition to flexion/extension. Importantly, the biarticular hamstring muscles were not only the hip extensors and knee flexors but also the hip adductors [11,12]. It is therefore plausible that the biarticular hamstring muscles are greatly lengthened in the hip abducted positions compared to the hip-neutral position in the hip flexed and knee extended positions. Supporting this, recent emerging evidence suggested that the shear modulus of BFlh was higher at 15° abduction of the hip joint than at 0° abduction with the hip and knee joints flexed to 70° and 0°, respectively [13]. Additionally, the shear moduli of ST and SM were higher at 45° abduction than at 0° abduction in the previous study [13]. As a larger acute decrease in passive muscle stiffness was observed after static stretching at long muscle lengths than that at short muscle lengths [14], eccentric-only resistance exercise in the hip abducted position could potentially induce a greater acute decrease in passive stiffness of the biarticular hamstring muscles. However, this possibility has not been tested yet.

This study aimed to investigate the effects of hip abducted position (0° abduction [ABD0], 20° abduction [ABD20], and 40° abduction [ABD40]) during eccentric-only knee flexion on acute changes in passive stiffness of the biarticular hamstring muscles. It was hypothesized that passive stiffness of the biarticular hamstring muscles would decrease more after eccentric-only knee flexion at ABD20 and ABD40, followed by ABD0 based on the previous finding [13].

## Materials and methods

### Participants

The priori power analysis for change in shear moduli of the biarticular hamstring muscles was conducted to determine the number of participants with a power of 80%, an α error of 0.05, and an effect size (Cohen’s *d*) of 0.95 using G*Power 3.1 software (Heinrich Heine University, Dusseldorf, Germany). The effect size of 0.95 was chosen according to the previous study that showed a significant acute decrease in the shear modulus of BFlh after repeated eccentric-only knee flexions [10]. The required minimal sample size was calculated to be 9. Thus, eleven healthy young male participants (169.7 ± 4.5 cm, 66.8 ± 9.0 kg, 25.5 ± 4.8 years; mean ± SD) were recruited for this experiment. These participants were recruited between April 1 and June 30, 2024. None of the participants had engaged in any specific sport events for at least 1 year prior to this study or had a history of neuromuscular diseases or musculoskeletal injuries of the right lower extremity in the past 5 years. They were required to refrain from strenuous exercise and alcohol consumption for 48 h before each experimental day. Throughout the experiment, the participants were instructed to maintain their daily activities and not to perform any additional exercises, such as stretching or resistance exercises. All participants provided written informed consent after receiving both written and verbal explanations of the study’s purpose, procedures, potential risks, and associated burdens. The present study was approved by the Ethics Committee of the authors’ institution (no. 23057) and was conducted according to the principles of the Declaration of Helsinki, except for the registration to database.

### Experimental design

**Fig 1:**
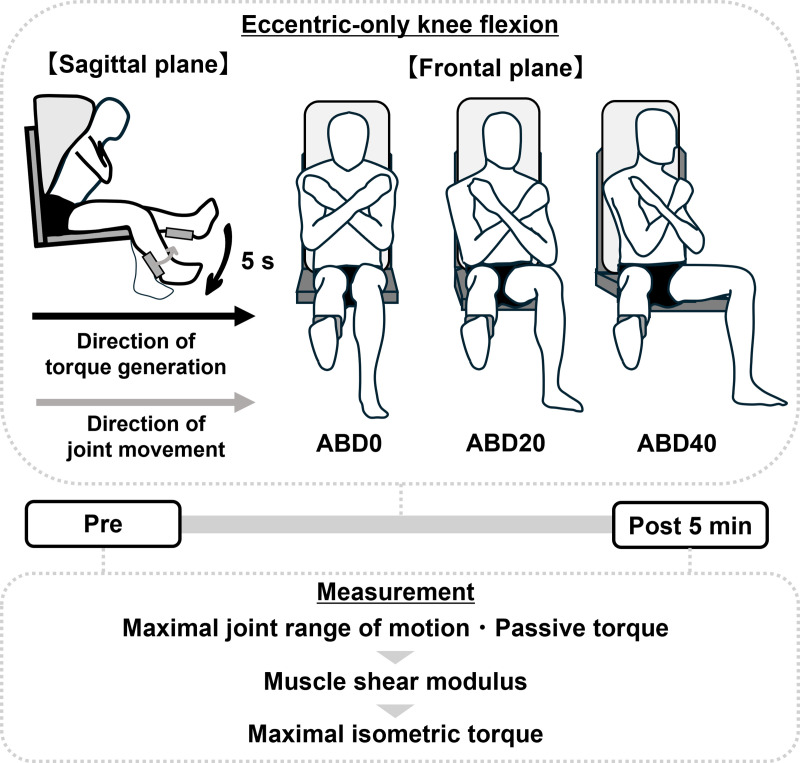
Overview of the present study design. **Participants performed three sessions of eccentric-only knee flexion over three days**. Maximal knee joint range of motion in a supine position, passive torque, shear moduli of the biarticular hamstring muscles, and maximal isometric torque of knee flexion were measured at pre and 5 min post-exercise in each session. ABD0, 0° abduction; ABD20, 20° abduction; ABD40, 40° abduction.

The present study adopted eccentric-only knee flexion based on a previous study reporting that passive knee extension stretching with the hip flexed immediately decreased the shear moduli of all biarticular hamstring muscles [8]. Participants completed a familiarization session on their first visit to our laboratory (Fig 1) considering the repeated bout effects (i.e., greater muscle damage on the first occasion than on the subsequent occasion [15]) on the results of the muscle shear modulus. On the subsequent 3 days (an interval of > 5 days), they completed one of the three resistance exercise sessions that consisted of eccentric-only knee flexion with different hip abducted positions: (1) ABD0, (2) ABD20, and (3) ABD40. The order of hip abducted positions was randomized across participants. Maximal knee joint range of motion (ROM) in the supine position, passive torque, shear moduli of the biarticular hamstring muscles, and maximal isometric torque of knee flexion were measured before and 5 min after completing each session in the order described above. In this study, we focused on measuring muscle shear modulus immediately after eccentric-only knee flexion based on a possible association between the immediate change in shear modulus induced by eccentric exercise and its chronic change [5,7]. However, the shear modulus was measured at 5 min after completing the session, due to the time required for post-exercise repositioning and setup. Some of the data at ABD0, such as the maximal joint ROM, passive torque, shear modulus, and maximal isometric torque, have also been used in another paper investigating the acute changes in passive stiffness of the biarticular hamstring muscles following repeated passive muscle lengthening and eccentric-only resistance exercise with different loads [16], but the present study expands upon the previous investigation by incorporating additional experimental conditions.

### Familiarization session

The familiarization session consisted of measuring maximal knee joint ROM and maximal eccentric torque of knee flexion in a seated position at ABD0, as well as practicing of eccentric-only knee flexions.

The participants were seated on a chair of an isokinetic dynamometer (Biodex System 4, Biodex Medical Systems, USA) with the hips flexed at 120° (full extension = 0°) and the right knee flexed at 90° (full extension = 0°). To maintain the hip flexion angle, a cushion was placed behind the back. The trunk, pelvis, and left thigh were securely fixed to the dynamometer bench using non-elastic straps. The rotation axis of the right knee joint was aligned with that of the dynamometer, and the right leg was attached to the lever arm. The knee joint was passively extended at an angular velocity of 2°/s from the initial position (90° of knee flexion) to the final position, where the participants started to feel discomfort and pressed the comfort stop button. The passive knee extension test was performed twice. The difference in the knee joint angle between the initial and final positions was defined as the maximal knee joint ROM in the seated position. The two measured values were averaged and used to determine the relative ROM during eccentric-only knee flexions. The joint angle and torque signals were sampled using an A/D converter (PowerLab 16SP, AD Instruments, Australia) at 1 kHz and then transferred to a computer.

To measure maximal eccentric torque, the exercise ROM was set from 50 to 100% of maximal knee joint ROM in the seated position. For example, when the maximal knee joint ROM ranged from 90° to 0°, 50% to 100% of the maximal ROM corresponded to 45° to 0° of knee flexion. The angular velocity was adjusted to either 5 or 10°/s, depending on each participant’s exercise ROM, ensuring that the exercise duration remained approximately 5 s. The exercise ROM and angular velocity were determined based on the previous studies [5,7]. The angular velocity of 5 or 10°/s during the exercise was chosen as follows. First, the exercise ROM was divided into 5 s. Then, the angular velocity was set to 10°/s when the calculated value was equal to or greater than 7.5°/s, and to 5°/s when it was less than 7.5°/s. This decision was made because of a limitation of the dynamometer, which did not allow angular velocities to be set between 5°/s and 10°/s.

As a warm-up, participants firstly completed five submaximal voluntary eccentric contractions of knee flexion while keeping their arms crossed over their chest. They then performed three maximal voluntary eccentric contractions (MVEC) of knee flexion with a 2 min rest period between trials. For each trial, the eccentric knee flexion torque was averaged over the exercise ROM. The highest mean value among the three trials was used to determine the target active torques at 50% of the maximal torque. This target torque level was used based on the previous studies showing immediate decreases in SM shear modulus after eccentric-only resistance exercise [5,7]. The target torque was calculated by taking into account the effects of gravity (the leg and lever arm) and passive torque determined during a passive knee extension test. The target torque was displayed to the participants on the monitor of a personal computer as real-time visual feedback. They performed 10 repetitions of eccentric-only knee flexion as practice.

### Main session

#### Resistance exercise.

Throughout the main sessions, the hip flexion angle, exercise ROM, target torque level, and angular velocity were set to be identical to those used in the familiarization session. The participants were seated on a handmade cushion specifically designed to prevent contact between the electrodes of electromyography (EMG) attached to the hamstring muscles (described in the following section) and the chair of the dynamometer. The hip abduction angle was defined as the change in angle between the horizontal line perpendicular to the line connecting both anterior superior iliac spines in the horizontal plane (reference axis) and the centerline of the right femur (moving axis). The hip abduction angle in each session was adjusted by rotating the chair of the dynamometer and then carefully confirmed using a manual goniometer. To minimize the motions of the hip internal/external rotation and pelvic anterior-posterior tilt during the exercise, both thighs and pelvis were tightly secured to the chair of the dynamometer as well as the trunk by using non-elastic straps. In each session, a single passive knee extension test at an angular velocity of 2°/s was performed over the exercise ROM for the corrections of gravity and passive torque. They then performed five repetitions of eccentric-only knee flexion at one of the hip abducted positions at 50% of MVEC torque with visual feedback as a warm-up. They performed MVEC of knee flexion once for the normalization procedure of EMG activity. Subsequently, they performed three sets of 10 repetitions of eccentric-only knee flexions, after confirming the absence of muscle contractions based on EMG signals. The target torque level and exercise ROM were 50% of MVEC torque and 50–100% of maximal knee joint ROM determined in the seated position at ABD0 during familiarization session, respectively. The angular velocity during eccentric-only knee flexions was identical to that during the familiarization session. The leg was passively repositioned between repetitions at the same angular velocity as that during the eccentric contraction. A rest of 2 min was allowed between sets to maintain the required torque levels throughout the session.

#### Maximal knee joint ROM and passive torque.

Participants lay in the supine position with their right hip flexed at 120° (full hip extension = 0°) and 90° of knee flexion (full knee extension = 0°). The rotation axis of the right knee joint was aligned with that of the dynamometer, and the right leg was attached to the lever arm. The knee joint was passively rotated at an angular velocity of 2°/s from the initial position (90° knee flexion) to the final position at which the participants started to feel discomfort and pressed the comfort stop button [8]. The passive knee extension test was performed once at each timepoint (pre and 5 min-post exercise). The change in the knee joint angle between the initial and final positions (i.e., maximal joint ROM in the supine position) was used as a measure of joint flexibility, and passive joint torque at the final position was used as a measure of stretch tolerance [17].

#### Muscle shear modulus.

A real-time ultrasound shear wave elastography system (Aixplorer Ver. 8, Supersonic Imagine, Aix-en-Provence, France) with a 4–15 MHz linear array transducer (SL15−4; Vermon, France) was used to quantify the passive stiffness of each biarticular hamstring muscle with MSK preset. The shear moduli of the biarticular hamstring muscles were measured at 80% (0% = 90°) of the maximal knee joint ROM evaluated in the supine position during the first exercise session based on the procedures of the previous studies [7,9]. This measurement position was chosen for two main reasons. First, a previous study reported that acute changes in shear modulus after eccentric exercise were detected at long muscle lengths, but not at short muscle lengths [18]. Additionally, another study showed that the relative acute decrease in shear modulus of SM appeared to be greater at the long muscle length (approximately −31.4% [calculated from mean value]) than at the short muscle length (approximately −18.9%), although those of BFlh and ST were comparable between two muscle lengths after one session of static stretching [6]. Second, another study showed that maintaining a muscle at an excessively long length during measurements induced an acute decrease in the shear modulus of the muscle [8]. In this position, the orientation of the transducer was adjusted to identify muscle fascicles of BFlh, ST, and SM within B-mode images obtained at 50%, 40%, and 60% of the thigh length (the distance between the greater trochanter [0%] and the popliteal crease [100%]), respectively [7,9]. The transducer position was marked with a permanent marker after carefully identifying several fascicles of each muscle. Based on the marked positions, static images of BFlh, ST, and SM were obtained twice within 2 min in a random order across participants at each timepoint. Each image was obtained after waiting approximately 5 s for the colormap in the elastogram to stabilize. The intra-day reliability of the shear moduli of the biarticular hamstring muscles was confirmed by 10 participants in our previous study [9] using the same procedures as this study. Briefly, the intra class correlation coefficients (1, 2) for the shear moduli of BFlh, ST, and SM were 0.95, 0.81, and 0.94, respectively. The coefficient of variations for the shear moduli of BFlh, ST, and SM were 2.6%, 1.9%, and 2.9%, respectively.

#### Maximal isometric torque.

The maximal isometric torque during knee flexion was measured at the same knee joint angle used for the elastography measurements (i.e., 80% of the maximal knee joint ROM). At each time point, the maximal isometric torque (3 s) was measured only once after five submaximal isometric contractions of knee flexion performed as a warm-up. During the test, participants grasped the bench with their hands and focused solely on performing knee flexion while avoiding hip extension. Vigorous encouragement was given to the participants to perform maximal voluntary isometric contraction (MVIC). The peak torque value during MVIC was defined as the maximal isometric torque.

#### EMG activity.

The surface EMG signals of the biarticular hamstring muscles were obtained during the measurements of shear modulus, as well as during isometric and eccentric contractions of knee flexion. After preparation of the skin by shaving the hair, rubbing with sandpaper, and cleaning with alcohol, pairs of pre-amplified surface electrodes (DL-141, S&ME, Tokyo, Japan, inter-electrode distance: 12 mm) were attached longitudinally at 55% of the thigh length for BFh, 45% for ST, and 65% for SM with surgical tape. A reference electrode was placed over the right patella. The EMG signals were simultaneously stored with the torque and joint angle at a sampling frequency of 1 kHz with band-pass filtering between 5 and 500 Hz (LabChart ver.8, AD Instruments, New South Wales, Australia). The root-mean square value of the EMG data (RMS–EMG) of each muscle was determined over 1.0 s period around the peak torque during MVIC of knee flexion at each timepoint. The RMS-EMG during shear modulus measurements were averaged across the two shear modulus measurements at each timepoint and normalized to that during MVIC before the resistance exercise as %MVIC. During eccentric contractions of knee flexion, RMS-EMG of the biarticular hamstring muscles was calculated over the entire exercise ROM, and the values were averaged over all repetitions. The RMS-EMG during eccentric contractions was then normalized to that during MVEC before the resistance exercise as %MVEC.

#### Statistical analysis.

The Shapiro-Wilk normality test was used to assess the distribution of the shear moduli of the biarticular hamstring muscles, and the results indicated that some of the data were non-Gaussian (*p* = 0.001 to 0.974). Hence, non-parametric tests were performed for all of the data in this study. The Friedman test was used to compare the measured variables (maximal knee joint ROM in the supine position, passive torque, shear moduli of the biarticular hamstring muscles, and maximal isometric torque of knee flexion) among the hip abducted positions at pre-exercise. The Friedman test was also performed to determine the significant effects of three hip abducted positions on the relative torque (torque in each session relative to the maximal voluntary eccentric torque measured during the familiarization session) and RMS-EMG of each muscle during eccentric-only knee flexion. When a significant main effect of hip abducted position was detected, the Wilcoxon signed-rank test was performed to identify significant differences in the relative torque and RMS-EMGs. The Wilcoxon signed-rank test was used to identify significant differences in the measured variables between timepoints in each session. The Friedman test was adopted to examine differences in the change values in the maximal knee joint ROM in the supine position, passive torque, shear moduli of the biarticular hamstring muscles, and maximal isometric torque of knee flexion between pre- and post-exercise among hip abducted positions.

The significant level was set at *p* < 0.05. For multiple tests, the *p* values were corrected based on the number of paired comparisons with Benjamini–Hochberg method [19] at a cutoff of a false discovery rate of < 0.05. The effect size (*r*) was calculated for all the tests in the present study and was interpreted according to the criteria proposed by Cohen [20], with |r| = 0.1, 0.3, and 0.5 representing small, medium, and large effects, respectively. All statistical analyses were performed using the statistical software package (IBM SPSS Statistics, version 29.0, IBM Corporation, Armonk, USA) and the web application (Langtest, Atsushi Mizumoto, Kansai University, Japan [Mizumoto and Plonsky, 2016]).

## Results

The individual data for the measured variables are shown in the supplementary information (S1 File). The Friedman test revealed no significant differences in all measured variables among three hip abducted positions at pre-exercise (corrected *p* = 0.227 to 0.913, *r* = 0.03 to 0.63).

**Fig 2:**
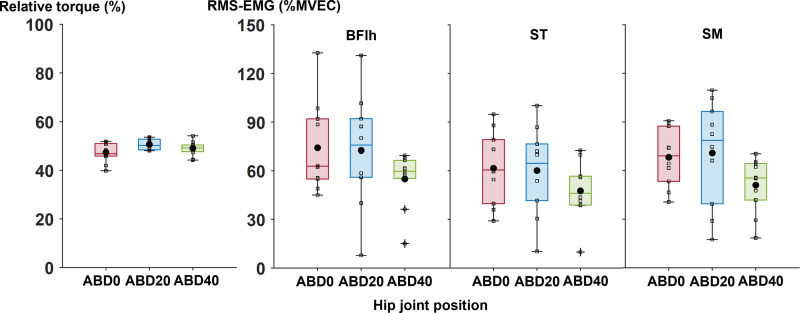
Boxplots of the relative torque to maximal voluntary eccentric torque and the root-mean-square of electromyograms (RMS-EMG) of the biarticular hamstring muscles in each session of eccentric-only knee flexion. Black-filled circles represent group mean values, while pluses indicate outlier values for each measured variable. The squares on each boxplot represent individual data points. MVEC, maximal voluntary eccentric contraction; ABD0, 0° abduction; ABD20, 20° abduction; ABD40, 40° abduction; BFlh, biceps femoris long head; ST, semitendinosus; SM, semimembranosus.

There were no significant main effects of hip abducted positions on the relative torque (corrected *p* = 0.156, *r* = 0.63) or RMS-EMG of BFlh (corrected *p* = 0.301, *r* = 0.33), ST (corrected *p* = 0.269, *r* = 0.40), or SM (corrected *p* = 0.269, *r* = 0.46 [Fig 2]) during eccentric contractions of knee flexion.

**Fig 3:**
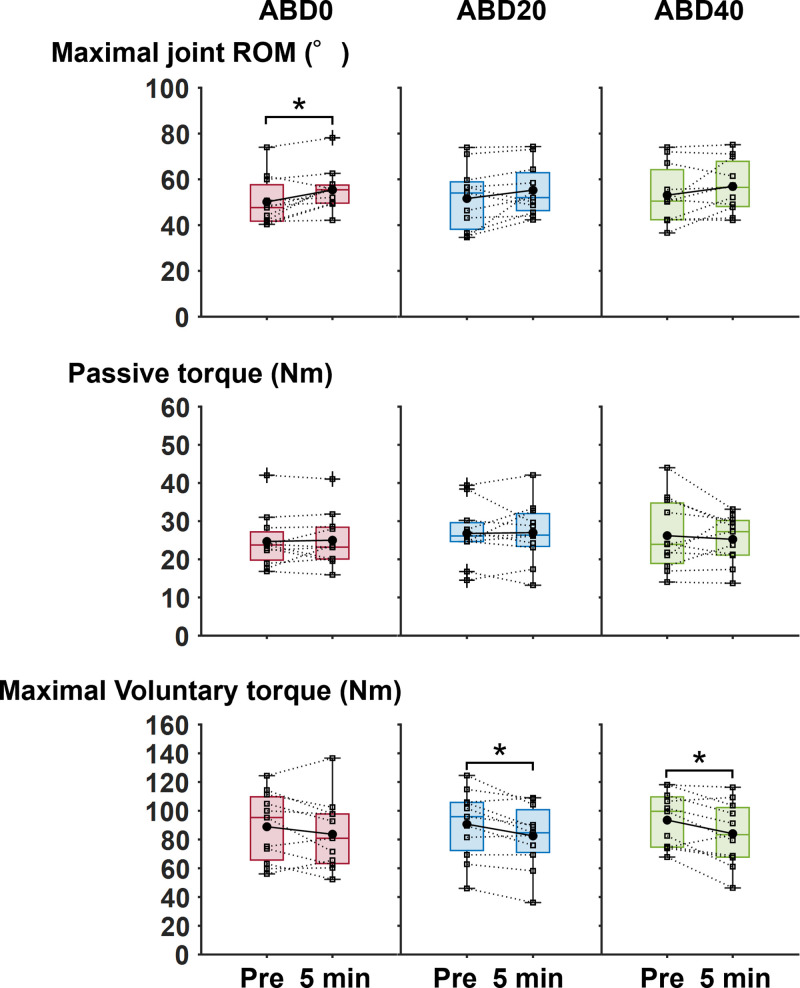
Boxplots of changes in maximal knee joint range of motion (ROM) in a supine position, passive torque, and maximal isometric torque at pre- and 5 min post-exercise in each session. Black-filled circles represent group mean values, while pluses indicate outlier values for each measured variable. The squares on each boxplot represent individual data points. *Significant difference between corresponding time points at *p* < 0.05. ABD0, 0° abduction; ABD20, 20° abduction; ABD40, 40° abduction.

The maximal knee joint ROM in the supine position was significantly wider at 5 min-post exercise than at pre-exercise at ABD0 (corrected *p* = 0.007, *r* = 0.59), whereas that was not significantly different between timepoints at ABD20 (corrected *p* = 0.152, *r* = 0.35) or ABD40 (corrected *p* = 0.206, *r* = 0.35 [Fig 3]). There were no significant differences in passive torque between pre-exercise and 5 min-post exercise in all hip abducted positions (corrected *p* = 0.824 to 0.966, *r* = 0.01 to 0.14). The maximal isometric torque of knee flexion was significantly lower at 5 min-post exercise than at pre-exercise at ABD20 (corrected *p* = 0.028, *r* = 0.55) and ABD40 (corrected *p* = 0.028, *r* = 0.50), but not at ABD0 (corrected *p* = 0.206, *r* = 0.27). There were no significant main effects of hip abducted positions on changes in the maximal knee joint ROM in the supine position (corrected *p* = 0.794, *r* = 0.31), passive torque (corrected *p* = 0.913, *r* = 0.03), or maximal isometric torque (corrected *p* = 0.794, *r* = 0.19).

**Fig 4:**
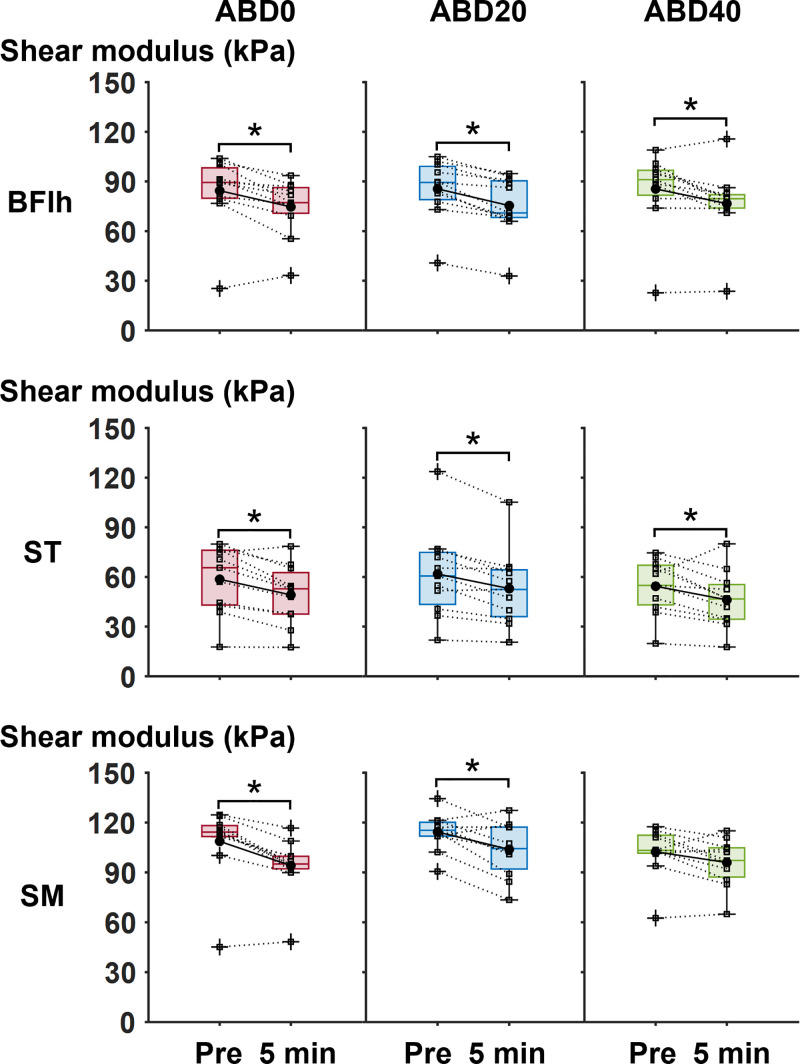
Boxplots of changes in the shear moduli of the biarticular hamstring muscles at pre- and 5 min post-exercise in each session. Black-filled circles represent group mean values, while pluses indicate outlier values for each measured variable. The squares on each boxplot represent individual data points. *Significant difference between corresponding time points at *p* < 0.05. ABD0, 0° abduction; ABD20, 20° abduction; ABD40, 40° abduction; BFlh, biceps femoris long head; ST, semitendinosus; SM, semimembranosus.

The shear moduli of BFlh and ST were significantly lower at 5 min post-exercise than at pre-exercise at ABD0 (corrected *p* = 0.004 to 0.021, *r* = 0.53 to 0.63), ABD20 (corrected *p* = 0.003 to 0.004, *r* = 0.63 to 0.70), and ABD40 (corrected *p* = 0.024 to 0.032, *r* = 0.46 to 0.48 [Fig 4]). The shear modulus of SM was significantly lower at 5 min post-exercise than at pre-exercise at ABD0 (corrected *p* = 0.006, *r* = 0.66) and ABD20 (corrected *p* = 0.021, *r* = 0.53), but not at ABD40 (corrected *p* = 0.083, *r* = 0.37). There were no significant main effects of hip abducted positions on changes in the shear moduli of BFlh (corrected *p* = 0.913, *r* = 0.33), ST (corrected *p* = 0.794, *r* = 0.19), or SM (corrected *p* = 0.794, *r* = 0.23). The median value of RMS-EMG during the shear modulus measurement throughout three sessions was 2.8%MVIC (0.1% to 4.8%MVIC [range from the minimum to the maximum values]) in BFlh, 2.8%MVIC (0.6% to 4.9%MVIC) in ST, and 2.7%MVIC (0.2% to 4.7%MVIC) in SM across all hip abducted positions.

## Discussion

This study revealed that the shear moduli of BFlh and ST decreased at 5 min post-exercise in all hip abducted positions. Additionally, the shear modulus of SM decreased at 5 min post-exercise at ABD0 and ABD20, but not at ABD40. There were no differences in the change in the shear moduli of the biarticular hamstring muscles among the hip abducted positions. Several studies have reported acute decreases in passive muscle stiffness after resistance exercise [7,9,10], but it remains unclear whether resistance exercise performed in a hip abducted position greatly and acutely decreases passive muscle stiffness. Thus, this is the first study to suggest that eccentric-only resistance exercise in the hip abducted positions does not necessarily amplify the acute decrease in the passive stiffness of the biarticular hamstring muscles.

At 5 min post-exercise, the shear moduli of BFlh and ST decreased in all hip abducted positions, and the shear modulus of SM decreased at ABD0 and ABD20 in the present study. However, there were no differences in the change in the shear moduli of the biarticular hamstring muscles among the hip abducted positions examined. These results did not support our hypothesis that passive stiffness of the biarticular hamstring muscles would decrease more after eccentric-only knee flexion at ABD20 and ABD40, followed by ABD0. One plausible explanation for these results is the minor influence of the hip adduction moment arm on acute changes in passive muscle stiffness. According to the anatomical study [11], in the biarticular hamstring muscles, the hip abduction moment arms (0.4 to 1.9 cm) were shorter than in the hip extension moment arms (4.6 cm to 5.6 cm) at 0° of hip flexion. Moreover, in another study, the hip adduction moment arm of BFlh appears to be shorter in the 45° hip flexed position (1.3 to 1.9 cm) than in the 0° hip flexed position (2.2 to 2.6 cm [12]). In the present study, the hip flexion angle was set at 120° during eccentric-only knee flexion to impose a large amount of lengthening on the biarticular hamstring muscles. In this hip flexion angle, the hip adduction moment arms of the biarticular hamstring muscles may be substantially short, and the amount of lengthening in the biarticular hamstring muscles may remain almost unchanged from ABD0 to ABD40. The short hip adduction moment arm in the hip flexed position may explain the lack of differences in the acute changes in passive stiffness across the hip abducted positions. Meanwhile, a previous study reported that muscle activation patterns of the biarticular hamstring muscles during eccentric-only resistance exercise could influence acute changes in passive muscle stiffness [21]. However, as shown in Fig 2, no significant differences in RMS-EMG of each biarticular hamstring muscle were observed among three hip abducted positions. These similar muscle activation levels during exercises may be a factor for explaining no differences in the change in the shear moduli of the biarticular hamstring muscles among the hip abducted positions examined.

Muscle damage has been suggested as one of important factors influencing immediate changes in passive muscle stiffness. Several studies have shown that muscle shear modulus increases immediately after repeated eccentric contractions [22,23], possibly due to severe muscle damage accompanied by perturbations in calcium homeostasis [24]. In the present study, the maximal isometric torque (an index of muscle damage [22]) was significantly lower at 5 min post-exercise than at pre-exercise at ABD20 and ABD40, but not at ABD0. Thus, the immediate decrease in shear moduli of the biarticular hamstring muscles may have been attenuated by muscle damage at ABD20 and ABD40, even if the hamstring muscles were more lengthened at these positions than at ABD0. Meanwhile, some studies have reported that muscle fatigue induced by repeated contractions can cause an immediate decrease in muscle shear modulus [25–28]. However, these studies consistently used isometric contractions, which elicit greater metabolic and neural fatigue compared with eccentric contractions [29]. Thus, it remains unclear whether the fatigue-induced decreases in muscle stiffness occur following eccentric-only contractions, which is future theme that should be investigated.

In addition to high muscle stiffness [3], narrow joint ROM has been identified as a contributing factor to an increased risk of musculoskeletal injuries [30]. In the present study, maximal knee joint ROM in the supine position did not increase at ABD20 or ABD40. Furthermore, the shear modulus of SM did not decrease immediately after eccentric-only knee flexion at ABD40. From a practical point of view, eccentric-only knee flexion performed in the abducted hip positions (ABD20 and ABD40) may have limited benefits in preventing musculoskeletal injuries. Meanwhile, at ABD0, the shear moduli of the three biarticular hamstring muscles decreased, and maximal knee joint ROM in the supine position increased. Thus, eccentric-only knee flexion performed at ABD0 could be more recommended to practitioners aiming to reduce the risk of musculoskeletal injuries. It is of great interest to determine whether eccentric-only knee flexions at ABD0 chronically leads to a decrease in passive stiffness of the biarticular hamstring muscles and an increase in joint ROM.

The present study has several limitations that should be considered. First, muscle shear modulus was assessed from only a single region within each biarticular hamstring muscle to minimize the total measurement time. A previous study reported the proximo-distal difference in the absolute change in shear modulus within SM after stretching exercise [31]. Thus, it remains uncertain whether our findings are applicable to other regions of the biarticular hamstring muscles. Second, the angular velocity during eccentric-only knee flexion was inconsistent among the participants, so that the exercise duration would be approximately 5 s. However, changes in the shear moduli of the biarticular hamstring muscles did not significantly differ between participants who performed knee flexion at an angular velocity of 5°/s or 10°/s in each hip abducted position (corrected *p* = 0.279 to 0.850, *r* = 0.06 to 0.40). Additionally, the statistical power for some variables might not be sufficient to detect significant paired differences between time points, likely due to the limited sample size. Considering this possibility, we have additionally performed a post hoc power analysis using the actual effect size (*r*), α error, and sample size for the Wilcoxon signed-rank test (matched pairs) in G*Power. The results showed that the actual statistical power ranged from 33% to 51% for BFlh shear modulus, from 28% to 44% for ST shear modulus, and from 37% to 71% for SM shear modulus. To minimize the risk of type I error, future studies with larger samples are warranted. Moreover, as the present study focused on limited population (young males without a history of musculoskeletal injury), the generalizability of the present findings to other populations remains unclear. Finally, the measurement time point was limited at pre- and 5 min post-exercise in the present study. A previous study investigated the time-course changes (up to 60 min post-exercise) in the shear moduli of the biarticular hamstring muscles and other variables (e.g., maximal isometric torque) following eccentric-only resistance exercise [9]. In that study, the shear modulus of SM was significantly lower at 3 min post-exercise compared with pre-exercise and 30 min and 60 min post-exercise, indicating that passive stiffness of a specific muscle decreases immediately after the exercise but gradually returns to baseline within 30 min. In addition, the maximal isometric torque was significantly lower at 60 min compared to the other timepoints, suggesting a time-dependent decrease in maximal isometric strength after eccentric-only resistance exercise. Based on these findings, both passive muscle stiffness and maximal isometric strength may change over time following eccentric-only resistance exercise, which would be an important topic for future investigation from a practical standpoint

## Conclusion

The purpose of this study was to investigate whether eccentric-only resistance exercise in hip abducted positions, rather than in the hip-neutral position, induces a greater acute decrease in the passive stiffness of the biarticular hamstring muscles. The shear moduli of BFlh and ST were significantly lower at 5 min post-exercise than at pre-exercise in all hip joint positions. In SM, the shear modulus was significantly lower at 5 min post-exercise than at pre-exercise at ABD0 and ABD20, but not at ABD40. In the three muscles, there were no significant differences in the changes in the shear moduli at 5 min post-exercise among the hip abducted positions. These findings suggest that eccentric-only resistance exercise in hip abducted positions does not necessarily amplify the acute decrease in the passive stiffness of the biarticular hamstring muscles and may even attenuate the decrease in stiffness of a specific muscle.

## Supporting information

S1 File Supplementary information. (XLSX)
